# Adoption of hooped-battens in cold-formed steel built-up columns for superior axial performance

**DOI:** 10.1038/s41598-024-55907-8

**Published:** 2024-03-02

**Authors:** A. Jamshid. Sadid, Mohammad Adil Dar, A. Fayeq Ghowsi, A. Cüneyt Aydın

**Affiliations:** 1 Civil Engineering Department, Atatürk University, Erzurum, Turkey; 2 Department of Civil and Structural Engineering, University of Sheffield, Sheffield, UK; 3 Faculty of Architecture, Istanbul Technical University, Istanbul, Turkey

**Keywords:** Civil engineering, Engineering

## Abstract

Previous research on cold-formed steel (CFS) battened columns has identified the critical factors influencing their performance and accordingly, their limiting values for improved performance have been recommended. However, these studies involved connecting battens to the chords (channels) via their flanges, leaving the slenderest component disconnected from the web. This study introduces a novel hooped-batten (tubular-element) that links both webs and flanges of the chords together, thereby improving the structural integrity of the built-up system and curtailing the half-wave buckling length in the webs. As a result, axial strength and stability in these built-up columns may improve adequately. Firstly, a numerical model of a conventional CFS battened column was developed in ABAQUS and verified against test results on the same reported in literature. Afterward, the validated model was used to simulate the behaviour of CFS built-up columns with hooped-battens. Two key parameters i.e., unbraced chord slenderness and overall column slenderness were varied to explore their influence on the axial behaviour of built-up columns in terms of peak strengths, failure modes and load-displacement characteristics. The performance of the hooped-battened columns was compared with the identical conventional battened columns, which reflects that the former exhibits superior strength and stability characteristics over the latter.

## Introduction

Due to advanced cold-forming machinery capable of manufacturing even the most intricate cross-sections with utmost precision, ease and speed, cold-formed steel (CFS) low to mid-rise structures are becoming popular in developed and developing countries^1–3^. The need to adopt built-up members arises when the single profiles fail to meet the capacity or stiffness requirements. Generally, hot-rolled steel built-up columns are constructed by adequately placing the chords transversely apart to achieve a higher radius of gyration, resulting in higher load-carrying capacities and stiffness responses. Contrary to this, in CFS construction, a conventional built-up column is fabricated by screw-fastening two channel-chords placed in the back-to-back arrangement through the web, and is mainly done due to ease in fabrication, despite possessing a far lesser efficiency than the closed sectional built-up profile as adopted in hot-rolled steel construction. Several studies have been carried out to extend this efficient approach to CFS structures by developing CFS built-up columns with closed sections and transversely spaced chords^4–19^. In some of these studies, plain angles were used as chords connected laterally using lacings or battens on all four sides^4,6,7,12^. The specimens with slender angle chords exhibited early buckling deformations during the primary stages of loading, which impacted their peak capacities. A better axial performance was achieved by adopting lipped angles instead of plain angles^9,10^. However, regardless of whether the angle chord is plain or lipped, this type of built-up construction involves a high density of fasteners along the column height, which imparts more geometric imperfections into the system, affecting its axial performance. Further, it requires more workforce for fabrication, as the lateral connectors need to be fastened on all four sides. This problem was conveniently resolved using channel chords, with lateral connections only on two sides^11,13–18^.

**Figure 1:**
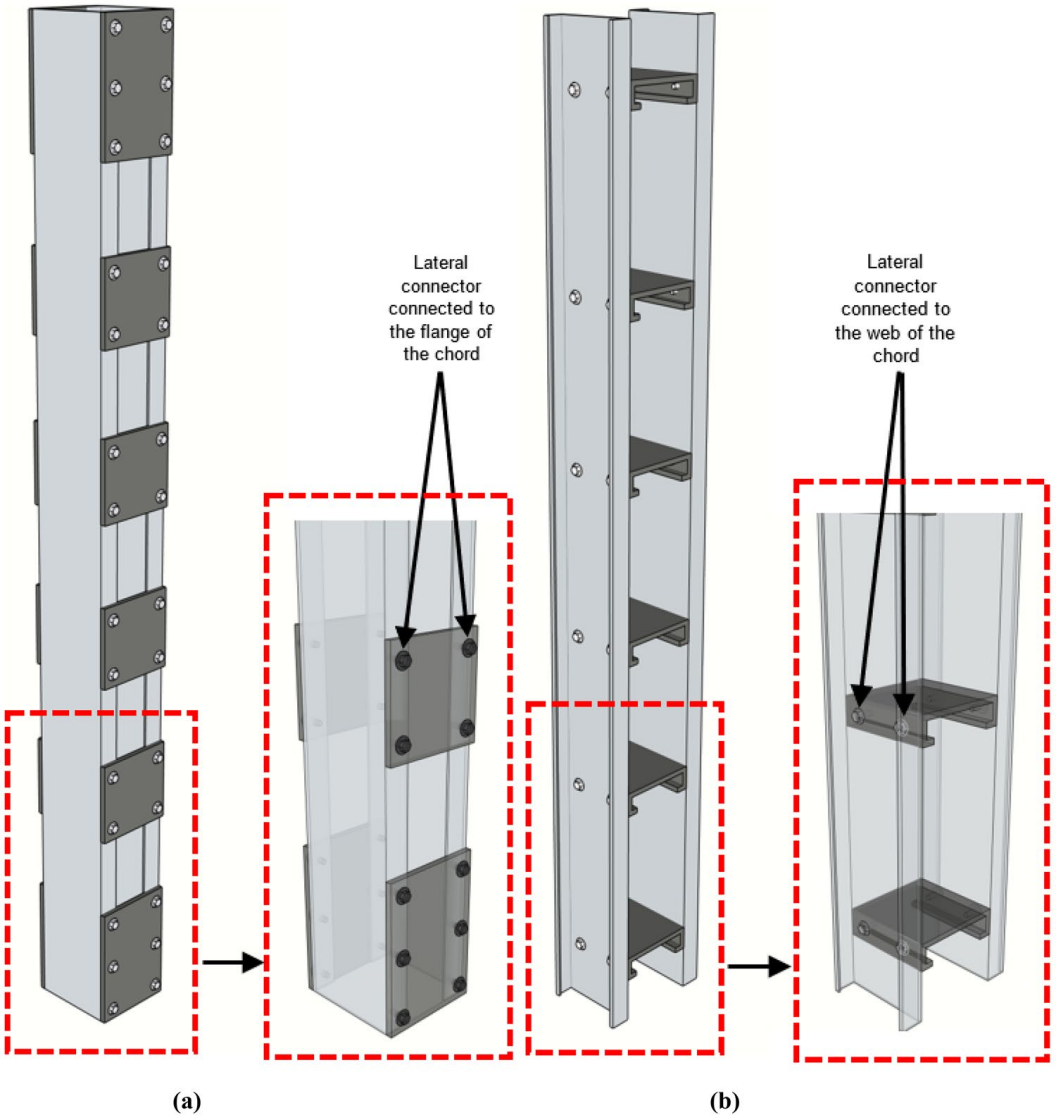
Connection details in a (**a**) Conventional battened column, (**b**) Built-up column with spacers.

**Figure 2:**
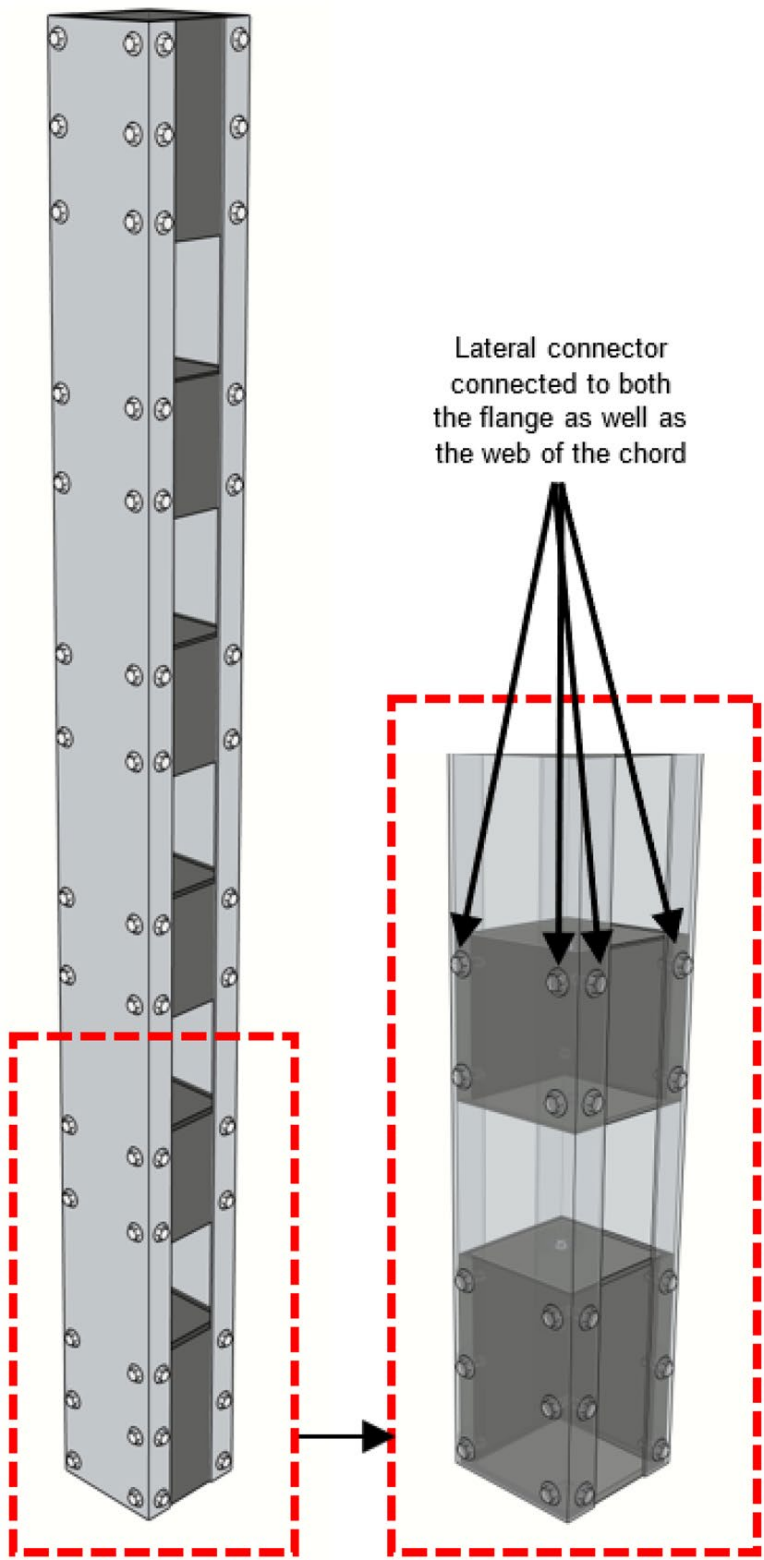
Connection details in hooped-battened columns.

Compared to laced columns, battened columns are way easier to fabricate, and through adequate design, higher axial capacity can be achieved with minimal material and fabrication costs. Various studies have been performed on CFS battened columns with channel chords, and variation in parameters like section-slenderness, built-up column slenderness, unrestrained chord slenderness (slenderness of the unrestrained chord between the intermediate battens), number of screws adopted per batten and sectional profile of the chord. In a recent study, an extensive investigation was performed on CFS battened columns with plain channel chords. Key parameters like section-slenderness, built-up column slenderness, unrestrained chord slenderness and relative slenderness of the unrestrained chord (ratio of unrestrained chord slenderness to built-up column slenderness) was carried out resulting in the development of the simplified design guidelines of such columns which are currently missing in the design standards^20,21^. The main problem with the conventional battened column system is that the battens are fastened to the channel chords through the flanges, despite the web being slenderer as shown in Fig. 1a. To address this issue, a few attempts were made to develop CFS built-up columns with channel spacers used as lateral connectors^22,23^. In this type of built-up system, the flanges of the spacer were connected to the webs of the channel chords, as shown in Fig. 1b. However, now the flanges are disconnected, and this type of built-up system did not catch much attention. To address this issue of either the web losing connection with the lateral connector or the flange, a new type of lateral connection namely hooped-batten (tubular-element) has been developed to ensure that both the webs as well as the flanges are adequately connected to the lateral connector (see Fig. 2), imparting more robust integrity in the built-up system. The new lateral connecting system will also curtail the half-wave buckling lengths in the flange and the web elements. This will significantly boost the axial resistance and stability capabilities in these built-up systems.

In the current paper a two-stage numerical study has been planned for CFS built-up columns connected laterally through novel hooped-battens. In the first step, a numerical model replicating a conventional CFS battened column system has been developed and calibrated against test results on similar system available in the literature. In the second step, the conventional battens were replaced by hooped battens in the verified numerical models to simulate the axial response of CFS built-up columns with hooped-battens. Variations in two of the most critical parameters i.e., unbraced chord slenderness and overall column slenderness were made to explore their effect on the axial characteristics of hoop-batten built-up systems on the basis of the peak axial strengths, failure modes and load-displacement behaviour. The axial response of the hooped-battened columns was compared against identical conventional battened columns, to quantify the performance enhancements achieved in the former.

### Numerical modelling technique adopted

**Figure 3:**
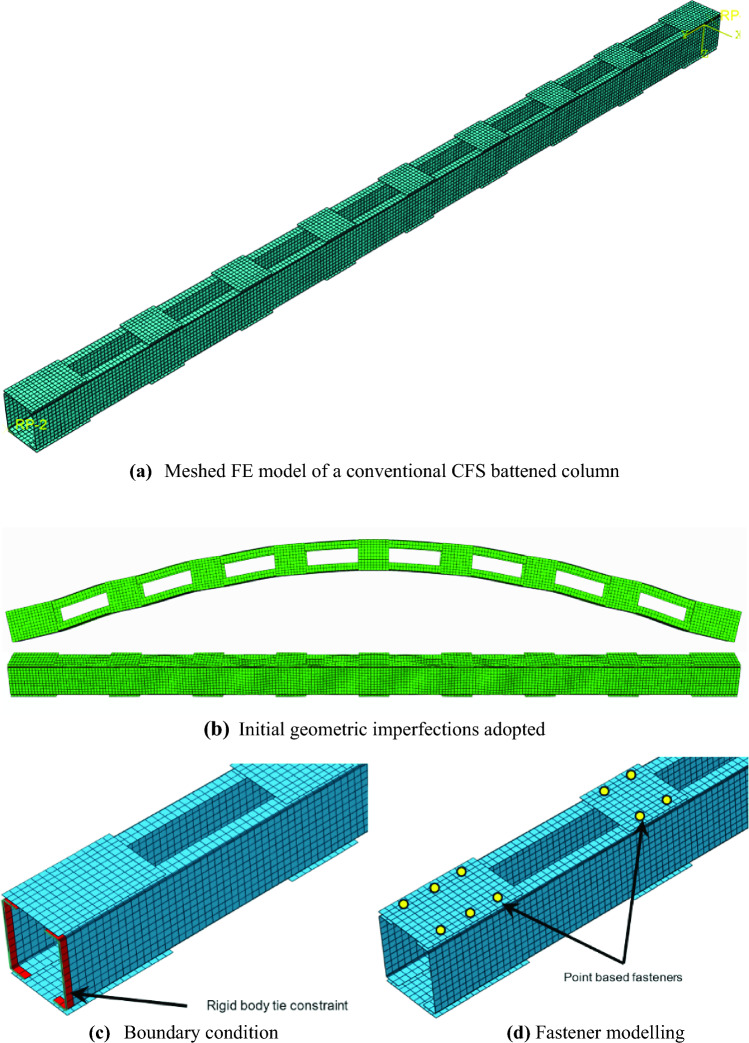
Details of FE modeling.

Numerical modelling is a viable and acceptable alternative when conducting tests is not feasible due to numerous constraints. ABAQUS^24^ is a commonly adopted commercial finite element software package used to conduct rigorous numerical studies, especially in structural engineering, and was also used in the current investigation. S4R type of shell elements are highly efficient in modelling thin-walled members and were accordingly used in simulating the chords and the battens. Adopting the right size and shape of mesh is essential in obtaining accuracy in numerical modelling without compromising on the computational efforts required to achieve the same. Therefore, a mesh convergence study was conducted on T2T-50-100^13^ by varying the square mesh size (5 mm, 10 mm, 15 mm, and 20 mm) to obtain the optimum size. The findings of the mesh convergence study are summarized in Table 1. Based on the results, a mesh size of 10 mm was chosen for flat regions to strike a balance between accuracy and computational efficiency. For the flange-web junctions, a finer mesh comprising of 3 elements was used. These selections align with similar approaches adopted in prior studies^11,13,14,16^. Figure 3a illustrates the meshed model of a conventional CFS battened column. The material of the chords as well as the battens was simulated using the average true stress *vs.* strain data presented in the test on CFS built-up battened columns reported in the literature^13,25^. As reported in previous studies, CFS thin-walled sections are highly sensitive to geometrical imperfections and need to be modelled adequately. On the recommendations of Schafer and Pecoz^26^ the local and global imperfections with an amplitude measuring 0.34 times the wall thickness of the chord and the built-up column/1000 were used respectively, as shown in Fig. 3b. Since the pin-ended boundary conditions were adopted in both the test series^13,25^, they were simulated as a rigid-body condition along with tie-constraints at both column ends (see Fig. 3c). The rigid-body constraints developed were connected to the reference points added to the geometrical centroid of the built-up section at both column ends. At the loaded end, to permit axial shortening in the loading direction and rotation of the built-up column in the direction parallel to the battens, these degrees of freedom were released and the others were restrained. A similar boundary condition was adopted at the other column end, except that the translation in the loading end was restricted. These boundary conditions at both the column ends were imparted to the respective reference points present there. When the battens are connected to the flanges of the chords, an overlap between the batten and the flange develops there. The surface-to-surface interaction at the overlapped region was modelled through hard contact with small sliding in the normal direction. The tangential contact with a frictional coefficient of magnitude of 0.19 in the tangential direction as recommended for galvanised steel surfaces^27^ was adopted. To mimic the screw fasteners adopted in battened tests^13^, point-based fasteners (3D beam connecting elements namely CONN3D2) were adopted (see Fig. 3d). On the contrary, the welded connection used in the battened tests^25^ was replicated using the edge-to-edge mesh-independent fasteners with all degrees of freedom restrained. The displacement-controlled monotonic axial loading as applied in the tests^13,25^ was imitated likewise at the loading end of the built-up column. Static general analysis was used in this current study to achieve the low-speed loading protocol as performed in the tests while accounting for the large buckling deformations in the chords of the CFS battened columns.

**Table 1:** Mesh convergence study results.

Mesh sizes (mm)	P_FEA_(kN)	P_Test_ (kN)	P_Test_/P_FEA_	Time (s)
5	181.02	176.15	0.97	3034
10	181.94		0.97	1876
15	182.34		0.97	1721
20	183.68		0.96	1522

### Verification of the numerical model and parametric study

**Figure 4:**
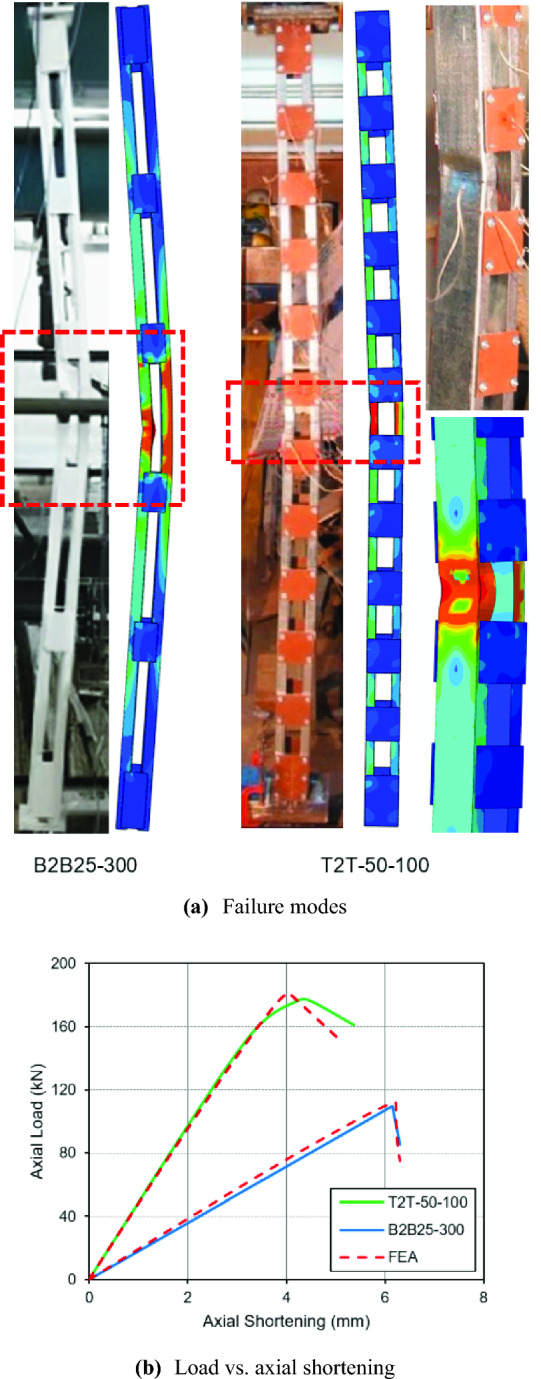
Validation of the FE model against CFS battened column test results. (**a**) Failure modes, (**b**) Load vs. axial shortening.

Before extending the numerical models to an extensive investigation, they need to be verified against test results adequately to impart a realistic simulation capability for achieving reliable outcomes. Therefore, the numerical models developed were calibrated against the two different sets of results available on CFS conventional battened columns reported^13,25^, both using plain channels as chords. The validation was performed against three characteristics namely peak axial loads, failure modes and load *vs.* axial shortening curves, for both sets of results, to achieve a higher reliability in the numerical models developed. The ratio of peak test capacities to their corresponding numerical capacities for both data sets^13,25^, with a mean and standard deviation are noted as 0.974 and 0.005, respectively, as presented in Table 2. The failure modes (Test *vs.* numerical) for specimens T2T-50-100 and B2B25-300 are illustrated in Fig. 4a. The load *vs.* axial shortening curves for specimens T2T-50-100 and B2B25-300 are shown in Fig. 4b. The comparison of the numerical behaviour against the same noted in the tests^13,25^ from Table 2, and Fig. 4 indicates that the numerical model is in good agreement with the test results and can be fit for the parametric study.

**Table 2:** Compression of FEA strengths with test strengths for battened column specimens.

Model	P_Test_ (kN)	P_FEA_(kN)	P_Test_/P_FEA_
T2T-50-100	176.23	181.94	0.97
T2T-50-175	157.41	161.96	0.97
T2T-100-175	163.01	167.72	0.97
B2B25-300	109.90	112.41	0.98
B2B75-300	125.30	127.24	0.98
		Mean	0.974
		Std. Dev.	0.005

The parametric numerical model is identical to the verified numerical model, except that the conventional battens are replaced with the hooped ones, as shown in Fig. 2. The cross-sectional aspect ratio of the parametric numerical models was fixed to unity, and the screw arrangements were extended from the test specimens of Dar et al.^13^, except that the screws continued in the web elements as well, as shown in Fig. 2. The thickness of the channel chord was adopted such that the sectional slenderness of the web is 50, which is within the limits, as per the recommendations of NAS^20^. The intermediate spacing between the hooped battens was adjusted such that the variation in the relative slenderness of the unbraced chord is achieved as 0.25, 0.50, 0.75 and 1.0. The overall slenderness values of the built-up hooped battened columns were varied from 10 to 180, with a regular interval of 10 in between. The length of the intermediate hooped batten was fixed at 100 mm and that of the end batten at ≥ 100 mm, both with a constant thickness of 6 mm. The variations in the intermediate batten spacing and the height of the columns resulted in 72 analyses. The labelling of the specimens was carried out carefully to reflect important parameters. For example, in HC-S50-0.50-60, HC represents Hooped-batten column, S50 indicates sectional slenderness of 50, 0.5 represents the ratio of unbraced chord slenderness to overall column slenderness, and the last term 60 reflects the overall slenderness of the built-up column.

## Results

To obtain conclusive outcomes, the numerical results were analyzed both in terms of the failure modes and load-axial shortening characteristics and will be discussed in the same order in this section. Since the overall slenderness of the hooped batten columns varied from 10 to 180, it covered all four categories of the columns based on their slenderness. In the previous studies on CFS battened columns with plain channel chords^16^, two distinct failure modes namely local buckling and global buckling and their interaction also known as interactive buckling were observed.

**Figure 5:**
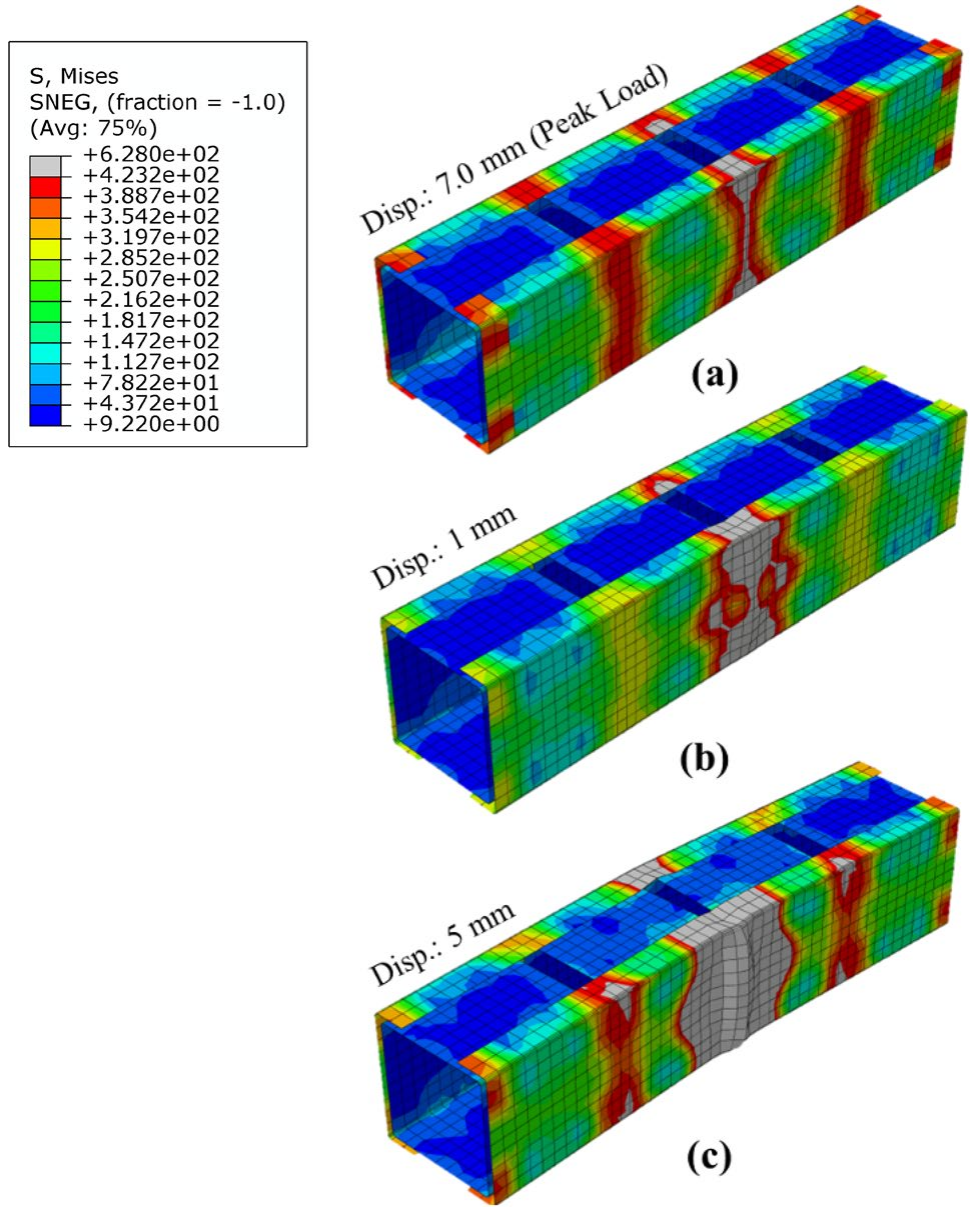
Failure progression in HC-S50-0.25-10.

**Figure 6:**
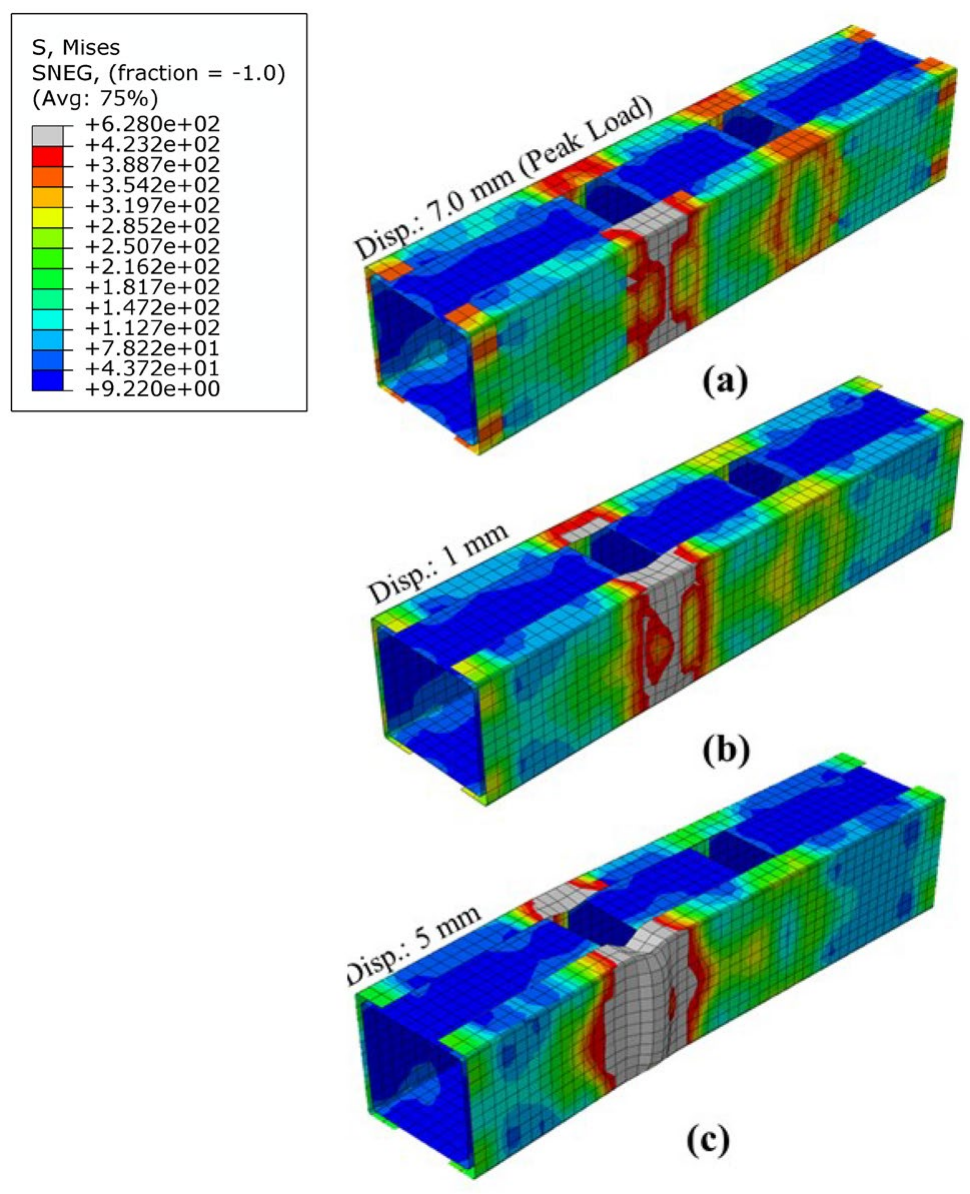
Failure progression in HC-S50-0.5-10.

As indicated in the previous study^16^, the battened columns with an overall column slenderness of 10 qualified as stub columns. In the current investigation, all four stub columns HC-S50-0.25-10, HC-S50-0.50-10, HC-S50-0.75-10, and HC-S50-1.00-10 failed by a combination of yielding and local buckling. At the peak load in HC-S50-0.25-10, yielding of the central unbraced panel was observed as shown in Fig. 5a. As the loading was increased further, the yielded portion started growing and additionally local bucking at the same location started emerging, as shown in Fig. 5b. On further progressing the loading, the yielded portion grew further which also extended to the flange-web junctions in other unbraced panels and the magnitude of the local buckling deformation started getting prominent in the central panel resulting in the final collapse of the column, as shown in Fig. 5c. In HC-S50-0.50-10, HC-S50-0.75-10, and HC-S50-1.00-10, a similar observation was noted, except that the yielded and locally bucking regions were not as dominant as were in HC-S50-0.25-10 and were limited to one panel only, as shown in Fig. 6 for HC-S50-0.50.

**Figure 7:**
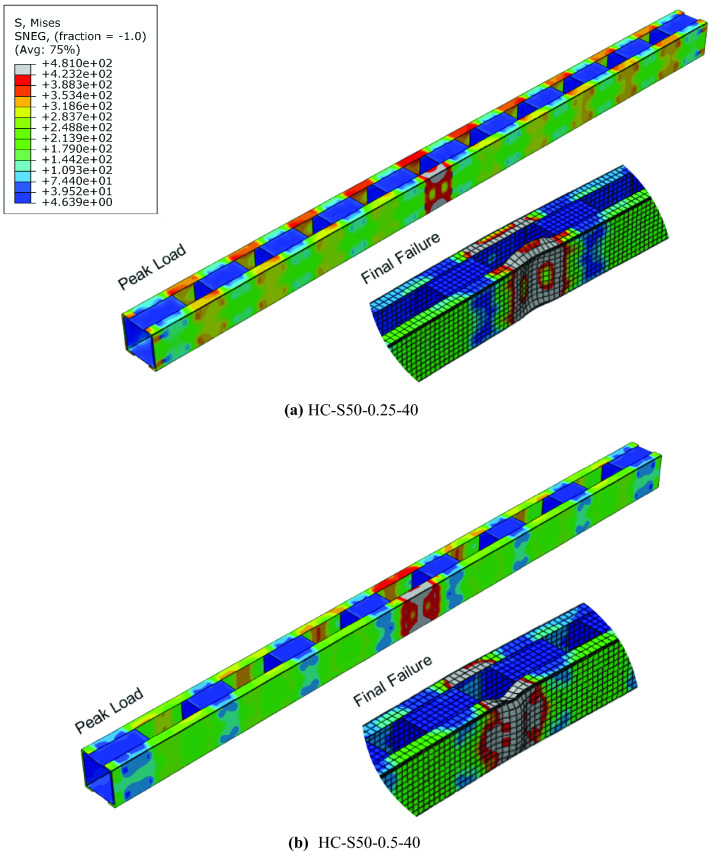
Failure modes in short columns.

All the short columns exhibited local buckling failure, as shown in Fig. 7a for HC-S50-0.25-40. It must be noted that although the other panel did not participate in the local buckling failure, the experienced higher levels of stress, as shown in stress contour in Fig. 7a. Specimens HC-S50-0.50-40, HC-S50-0.75-40, and HC-S50-1.00-40, also failed in the same manner, except that the stresses in the other panels was lower compared to HC-S50-0.25-40 as shown in stress contour in Fig. 7b for specimen HC-S50-0.50-40.

**Figure 8:**
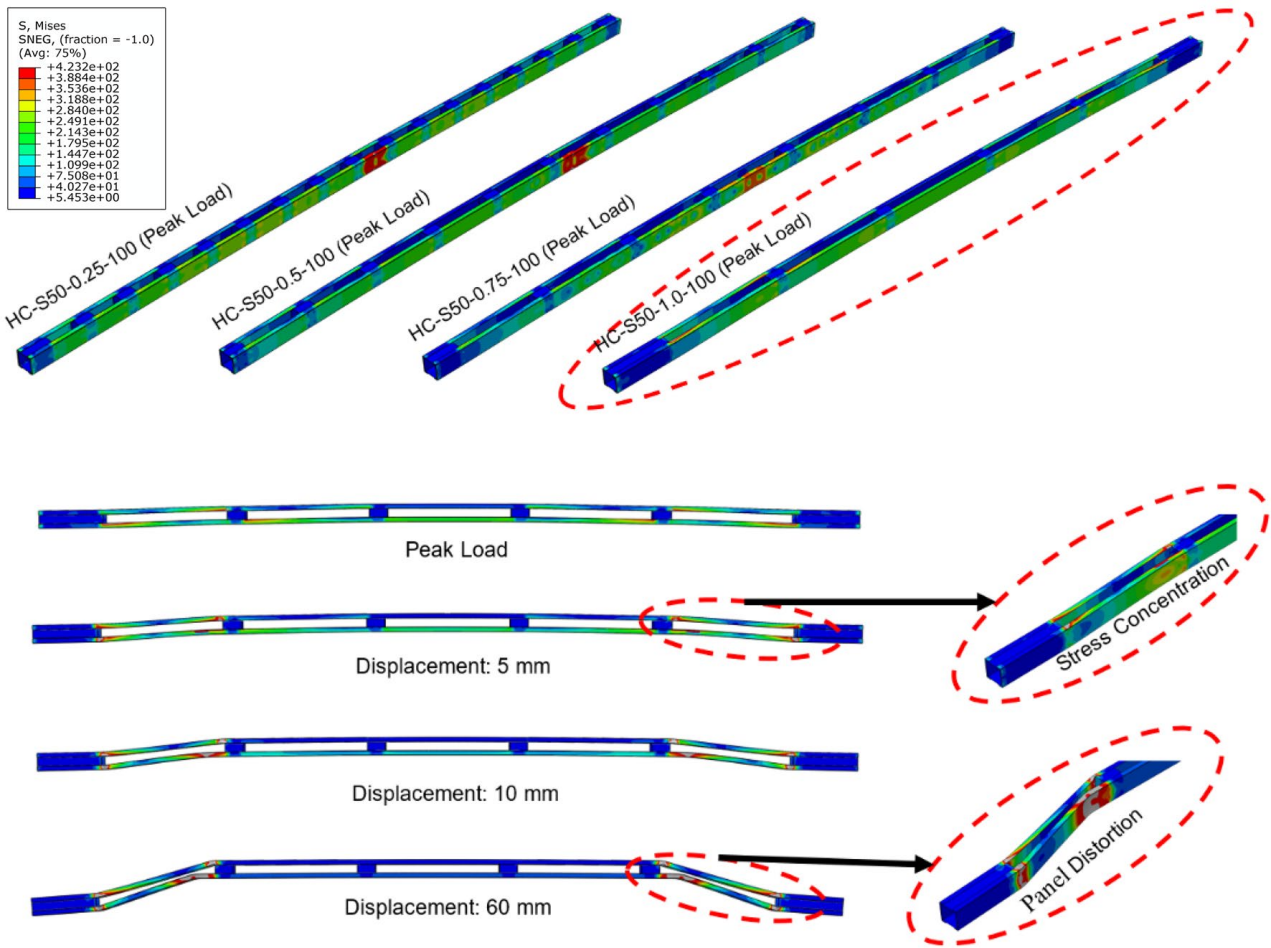
Failure modes in intermediate columns.

**Figure 9:**
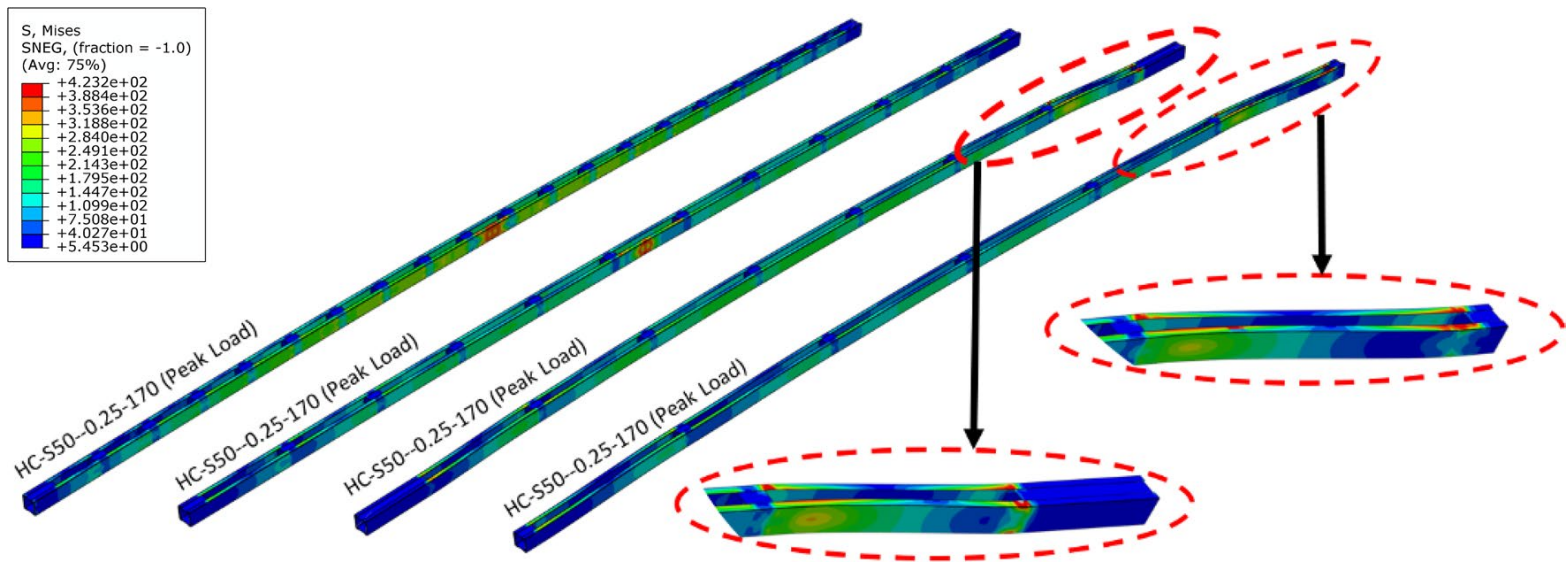
Failure modes in long columns.

In the intermediate columns, the failure observed was a combination of local and global buckling, collectively referred to as interactive buckling. In HC-S50-0.25-100, the magnitude of local buckling component was higher, as shown in Fig. 8, as it was the first intermediate column after the short columns concluded. Rest of the behaviour was identical to HC-S50-0.25-40, except that minor global buckling was also noted here. In HC-S50-0.50-100 and HC-S50-0.75-100, a behaviour similar to HC-S50-0.25-100 was noted, except lower magnitude of stress in the other panels, as shown in Fig. 8. In specimen HC-S50-1.00-10, distortion in the end unbraced panels was noted, as shown in Fig. 8, primarily due to weak structural integrity between the chords. All the long columns experienced global buckling as shown in Fig. 9 for HC-S50-0.25-170 and HC-S50-0. 50-170. Long specimens HC-S50-0.75-170 and HC-S50-1.00-170 failed by end panel distortion similar to intermediate column HC-S50-1.00-10, for the same reason stated earlier.

**Figure 10:**
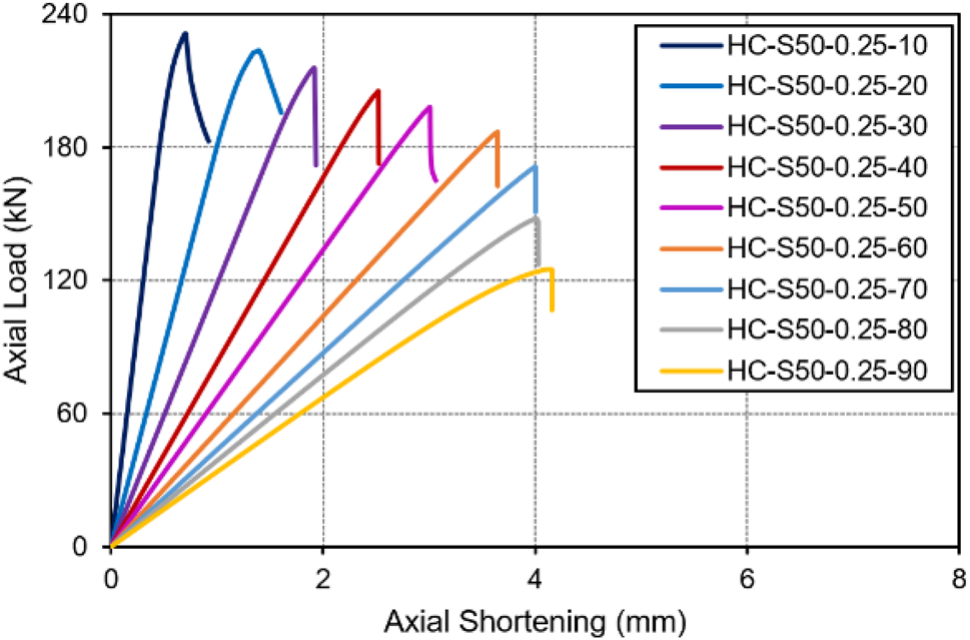
Load vs. axial shortening curves of stub and short columns.

The load-axial displacement characteristics of columns provide valuable insights into their behaviour, offering a glimpse into their behaviour by examining the plotted data. In Fig. 10, we can observe the load-axial shortening curves for a series of hooped batten columns (from HC-S50-0.25-10 to HC-S50-0.25-90), each having a relative unbraced chord slenderness of 0.25, and an overall slenderness ranging from 10 to 90. It must be noted that all these columns exhibited a consistent pattern in their load-displacement response. This behaviour remained linear until they reached their maximum axial loads, as illustrated in Fig. 10. The linear trend leading up to the peak loads is indicative of a local buckling failure. Beyond this point, there was a sudden drop in load observed across all the columns, which aligns with our observations of the failure mode in these specimens. This data substantiates the actual failure mode witnessed in these column specimens, as discussed previously.

All the stub and short hooped batten columns examined in this study displayed a uniform load-displacement behaviour. After carefully reading Fig. 10, it can be concluded that the hooped batten columns with overall slenderness values from 20 to 90, can be categorized as short columns based on their performance characteristics.

**Figure 11:**
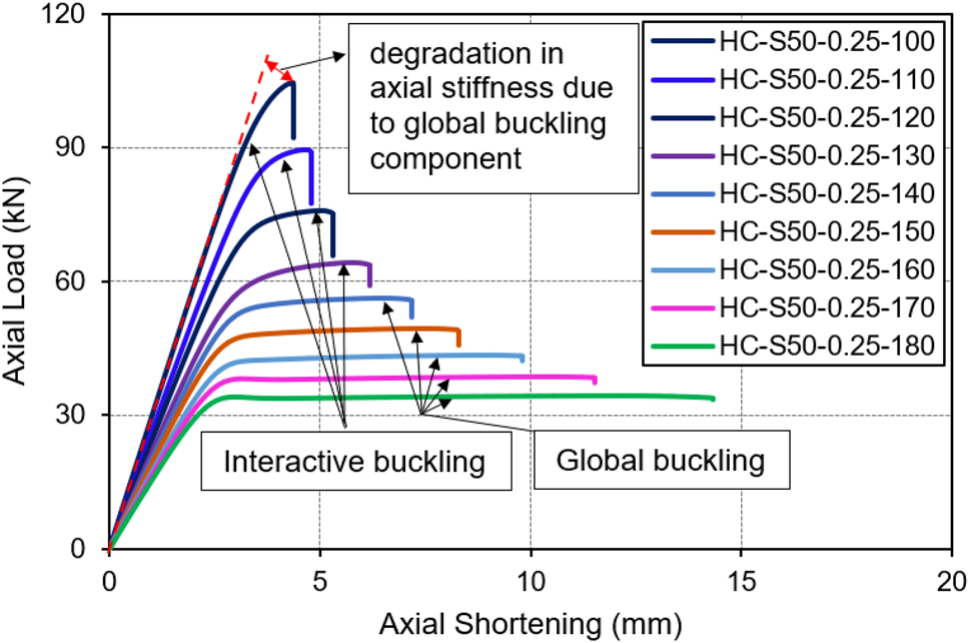
Load vs. axial shortening curves of intermediate and long columns.

Figure 11 presents the load-axial displacement curves for the same set of columns with the overall slenderness spanning from 100 to 180. In HC-S50-0.25-100, the initial part of the curve’s rising branch is linear till 90 kN (which is approximately 90% of its peak axial load) and resembles that of the short columns discussed previously. However, post 90 kN, the specimen starts experiencing a gradual reduction in its axial stiffness till the specimen reaches its peak load of 104 kN, indicating the presence of global buckling component. Following the peak load, a sudden drop akin to that in short columns is evident, suggesting a mixed behavior resulting from the interaction of global and local failure, with the latter following the former. Specimens HC-S50-0.25-110, HC-S50-0.25-120, and HC-S50-0.25-130 exhibited a similar behavior pattern, and as such, all four of these specimens are aptly categorized as intermediate columns. It is notable that HC-S50-0.25-100 exhibited a higher local buckling component compared to HC-S50-0.25-130, whereas the inverse holds true for the global buckling component. Specimens from HC-S50-0.25-140 onwards displayed a substantial prevalence of global buckling, denoted by the distinct flat horizontal portion of the curves in Fig. 11. These columns collapse only after displaying a significant amount of axial deformation, clearly indicating the presence of substantial global buckling. Accordingly, these specimens are categorized as long columns.

### Performance comparison (Hooped batten columns vs. Conventional batten columns)

**Figure 12:**
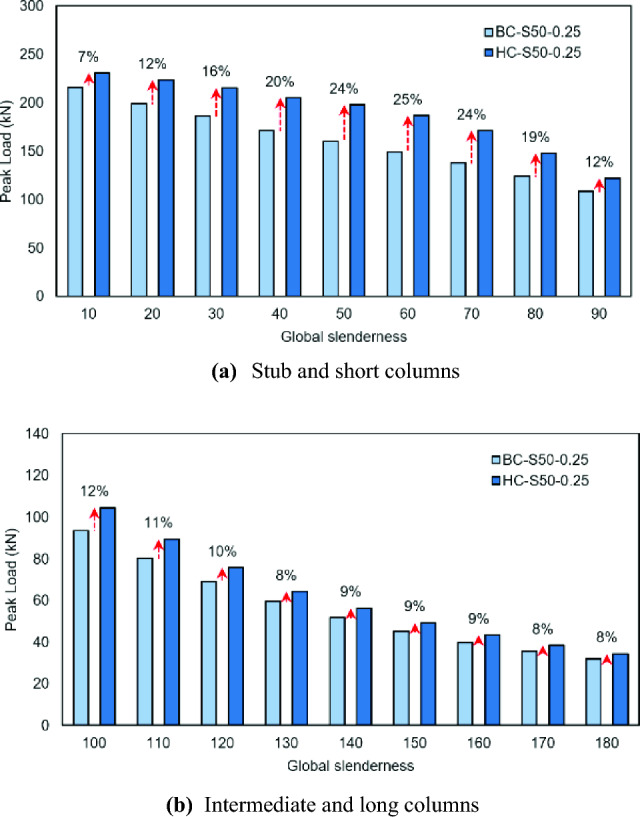
Comparison of peak load in conventional battened columns and hooped battened columns with relative unbraced slenderness values of 0.25.

**Figure 13:**
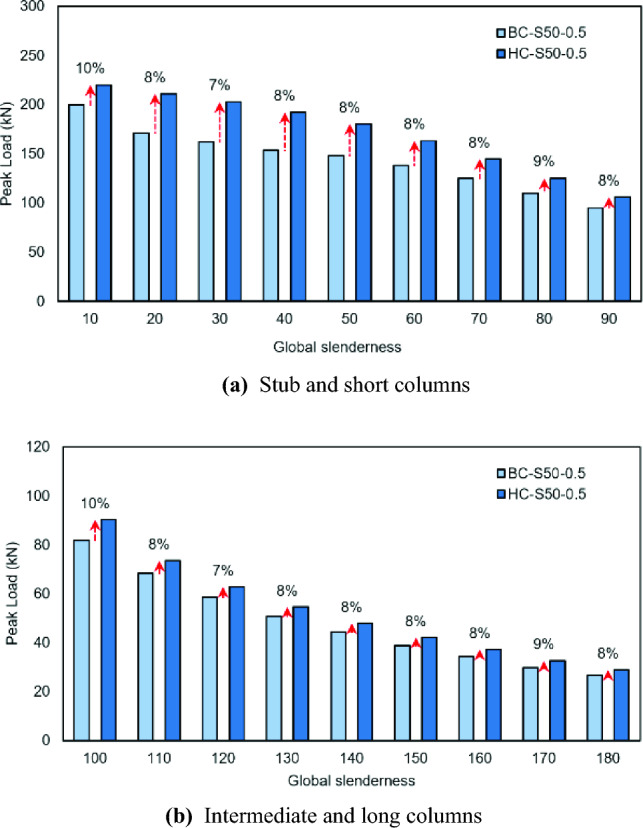
Comparison of peak load in conventional battened columns and hooped battened columns with relative unbraced slenderness values of 0.5.

The primary objective behind developing hooped batten columns was to achieve a higher axial capacity compared to conventional battened columns^16^. Accordingly, it is important to quantify the gains made in the former. The peak axial strengths of the hooped batten columns analyzed in this study have been compared with their corresponding columns (with the same overall slenderness, sectional slenderness, and unbraced chord’s slenderness) connected laterally with conventional battens reported previously^16^, as shown in Table 3, Figs. 12 and 13. In the stub column category with relative unbraced slenderness values of 0.25 and 0.5, the hooped batten columns carried a higher load than the conventional batten columns by 7% and 10% respectively. The gains when the relative unbraced slenderness values were 0.75 and 1.00 were noted as 17% in both and were attained mainly by engaging a larger part of the chord by using hooped battens. In the short column category, the maximum and minimum gains made were recorded as 25% and 7%, respectively in HC-S50-0.25-60 and HC-S50-1-90. In the intermediate column category, the maximum enhancements achieved were 12% and 7%, respectively in HC-S50-0.25-100 and HC-S50-0.5-120. In the long columns, the maximum and minimum gains did not vary much as both measured around 9%. Clearly, in the hooped batten columns the involvement of the web in addition to the flange resulted in mobilizing a larger part of the chord during the axial resistance development at the system level.

**Table 3:** Strength improvement of hopped battened columns compared to conventional battened columns.

SI.No	RSUC^a^- OS^b^	Column type	P_BC_^16^(kN)	P_HC_ (kN)	P_HC_ (kN)/P_BC_^16^(kN)
1	0.25-10	Stub	215.95	231.03	1.07
2	0.25-20	Short	199.15	223.78	1.12
3	0.25-30	Short	186.22	215.64	1.16
4	0.25-40	Short	171.51	205.43	1.20
5	0.25-50	Short	160.04	198.06	1.24
6	0.25-60	Short	149.15	186.71	1.25
7	0.25-70	Short	138.08	171.34	1.24
8	0.25-80	Short	124.27	147.77	1.19
9	0.25-90	Short	108.85	122.12	1.12
10	0.25-100	Intermediate	93.68	104.65	1.12
11	0.25-110	Intermediate	80.43	89.50	1.11
12	0.25-120	Intermediate	69.05	75.79	1.10
13	0.25-130	Intermediate	59.71	64.31	1.08
14	0.25-140	Long	51.82	56.32	1.09
15	0.25-150	Long	45.31	49.28	1.09
16	0.25-160	Long	39.93	43.51	1.09
17	0.25-170	Long	35.55	38.51	1.08
18	0.25-180	Long	31.87	34.44	1.08
19	0.5-10	Stub	199.90	220.18	1.10
20	0.5-20	Short	170.99	210.99	1.23
21	0.5-30	Short	162.17	202.86	1.25
22	0.5-40	Short	153.61	192.23	1.25
23	0.5-50	Short	147.71	180.35	1.22
24	0.5-60	Short	137.65	163.18	1.19
25	0.5-70	Short	124.96	144.70	1.16
26	0.5-80	Short	110.30	125.21	1.14
27	0.5-90	Short	95.23	106.20	1.12
28	0.5-100	Intermediate	81.72	90.25	1.10
29	0.5-110	Intermediate	68.39	73.59	1.08
30	0.5-120	Intermediate	58.63	62.83	1.07
31	0.5-130	Intermediate	50.79	54.60	1.08
32	0.5-140	Long	44.22	47.93	1.08
33	0.5-150	Long	38.85	42.00	1.08
34	0.5-160	Long	34.35	37.15	1.08
35	0.5-170	Long	29.81	32.57	1.09
36	0.5-180	Long	26.68	28.82	1.08
37	0.75-10	Stub	179.44	210.13	1.17
38	0.75-20	Short	159.76	190.52	1.19
39	0.75-30	Short	156.83	188.85	1.20
40	0.75-40	Short	149.11	181.18	1.22
41	0.75-50	Short	138.60	163.18	1.18
42	0.75-60	Short	127.34	149.53	1.17
43	0.75-70	Short	113.93	125.87	1.10
44	0.75-80	Short	96.88	105.08	1.08
45	0.75-90	Short	77.57	83.92	1.08
46	0.75-100	Intermediate	64.94	71.68	1.10
47	0.75-110	Intermediate	54.86	60.10	1.10
48	0.75-120	Intermediate	46.69	50.74	1.09
49	0.75-130	Intermediate	40.35	43.84	1.09
50	0.75-140	Long	35.21	38.29	1.09
51	0.75-150	Long	30.95	33.55	1.08
52	0.75-160	Long	27.45	29.99	1.09
53	0.75-170	Long	24.46	26.81	1.10
54	0.75-180	Long	21.84	23.66	1.08
55	1.00-10	Stub	173.72	203.97	1.17
56	1.00-20	Short	158.47	186.01	1.17
57	1.00-30	Short	152.26	180.05	1.18
58	1.00-40	Short	143.17	170.90	1.19
59	1.00-50	Short	133.17	156.47	1.17
60	1.00-60	Short	112.89	132.01	1.17
61	1.00-70	Short	92.62	99.70	1.08
62	1.00-80	Short	75.51	81.10	1.07
63	1.00-90	Short	62.28	66.77	1.07
64	1.00-100	Intermediate	52.13	57.40	1.10
65	1.00-110	Intermediate	43.89	48.02	1.09
66	1.00-120	Intermediate	37.51	40.84	1.09
67	1.00-130	Intermediate	32.19	35.27	1.10
68	1.00-140	Long	28.04	30.71	1.10
69	1.00-150	Long	24.69	26.87	1.09
70	1.00-160	Long	21.89	24.06	1.10
71	1.00-170	Long	19.45	21.07	1.08
72	1.00-180	Long	16.13	17.52	1.09

^a^Relative slenderness of unbraced chord.

^b^Overall slenderness.

## Conclusions

In this paper, a novel hooped batten in the form of a tubular-element was developed to connect the web as well as the flange elements to the chords, aiming to enhance the structural integrity of the built-up columns, primarily by effective curtailment of the chord’s half-wave buckling length. This exercise was carried out to improve the structural strength and axial stability in these built-up columns. In the beginning, a conventional CFS battened column was simulated in ABAQUS and then rigorously verified against its test results published in the literature. Later, the validated numerical model was used to replicate the axial response of CFS hooped batten column by simply changing the batten elements. Unbraced chord slenderness and overall column slenderness were altered and their effect on the peak strengths, failure modes and load-displacement curves were studied. It was noted that stub columns failed by yielding in the chord followed by local bucking while the short columns mainly failed by pure local buckling of the chords. The intermediate columns experienced interactive buckling while the long columns exhibited global buckling. In intermediate and long columns with relative slenderness of the unbraced chords exceeding 0.75, distortion in the end unbraced panels were noted. Lastly, the axial strengths of the hooped batten columns were compared with the conventional batten columns and capacity gains of as high as 25% were recorded in the former. These gains were a result of improved structural integrity engaging a larger part of the chord in the load distribution when compared to the conventional batten columns.

### Recommendations, limitations and future scope

From the findings of the current study, the following recommendations are proposed:

- Hooped battens should be adopted over conventional battens for improved structural performance due to superior structural integrity.
- The relative slenderness of the unbraced chord in CFS hooped batten column must not exceed 0.5 to avoid panel distortion that may otherwise occur in the end panels and significantly reduce the column capacity.
- Four screws on each face of the column for the intermediate hooped battens and six screws for the end hooped battens must be adopted to develop adequate integrity among the various components connected.

The presented recommendations are limited to CFS hooped batten columns comprising plain channels, with the dimensions and material properties of the specimens adopted in the current study. Hooped batten columns will require more fabrication time than the conventional I-type built-up columns. CFS hooped batten columns may not be suitable for the strapped stud wall system.

Further, this study can be extended by varying the thickness of the chord, exploring the influence of smaller increments of the unbraced chord’s relative slenderness, adopting composite chords, varying the size of screws, etc. The authors plan to extend this parametric study over a wider range of varying chord thicknesses and generate a large pool of data points. The numerical strengths will be compared against the design strengths based on the current design codes to assess their adequacy. Suitable design proposals will be brought out if needed.

## Data Availability

The datasets used and/or analyzed during the current study available from the corresponding author on reasonable request.

## References

[1] Beatini V, Gatheeshgar P, Rajanayagam H (2022). Integration of origami and deployable concept in volumetric modular units. Sci. Rep..

[2] Obst M, Wasilewicz P, Adamiec J (2022). Experimental investigation of four-point bending of thin walled open section steel beam loaded and set in the shear center. Sci. Rep..

[3] Hou H, Chen Z, Wang X (2023). Experimental study on the seismic performance of a cold-formed thin-walled steel–concrete composite column-H steel beam frame. Sci. Rep..

[4] El Aghoury MA (2010). Experimental investigation for the behaviour of battened beam-columns composed of four equal slender angles. Thin-Walled Struct..

[5] Aghoury EI, MA, Salem AH, Hanna MT, Amoush EA.  (2013). Ultimate capacity of battened columns composed of four equal slender angles. Thin-Walled Struct..

[6] Dar MA, Sahoo DR, Pulikkal S, Jain AK (2018). Behaviour of laced built-up cold-formed steel columns: Experimental investigation and numerical validation. Thin-Walled Struct..

[7] Dar MA, Sahoo DR, Jain AK (2019). Axial compression behavior of laced cold-formed steel built-up columns with unstiffened angle sections. J. Constr. Steel Res..

[8] Kherbouche S, Megnounif A (2019). Numerical study and design of thin walled cold formed steel built-up open and closed section columns. Eng. Struct..

[9] Anbarasu M (2020). Behaviour of cold-formed steel built-up battened columns composed of four lipped angles: Tests and numerical validation. Adv. Struct. Eng..

[10] Anbarasu M, Dar MA (2020). Improved design procedure for battened cold-formed steel built-up columns composed of lipped angles. J. Constr. Steel Res..

[11] Dar MA, Sahoo DR, Jain AK (2020). Influence of chord compactness and slenderness on axial compression behavior of built-up battened CFS columns. J. Build. Eng..

[12] Dar MA, Sahoo DR, Jain AK (2021). Interaction between chord compactness and lacing slenderness in CFS built-up columns. Structures.

[13] Dar MA, Sahoo DR, Jain AK, Sharma S (2021). Monotonic tests and numerical validation of cold-formed steel battened built-up columns. Thin-Walled Struct..

[14] Rahnavarad R, Craveiro HD, Laím L, Simões RA, Napolitano R (2021). Numerical investigation on the composite action of cold-formed steel built-up battened columns. Thin-Walled Struct..

[15] Dar MA, Subramanian N, Anbarasu M, Ghowsi AF, Arif PA, Dar AR (2021). Testing and FE simulation of lightweight CFS composite built-up columns: Axial strength and deformation behavior. Thin-Walled Struct..

[16] Dar MA, Verma A, Anbarasu M, Pang SD, Dar AR (2022). Design of cold-formed steel battened built-up columns. J. Constr. Steel Res..

[17] Dar MA, Sahoo DR, Jain AK, Verma A (2022). Tests on CFS laced columns composed of plain channels: Behavior and design. J. Struct. Eng.-ASCE.

[18] Rahnavard R, Razavi M, Fanaie N, Craveiro HD (2023). Evaluation of the composite action of cold-formed steel built-up battened columns composed of two sigma-shaped sections. Thin-Walled Struct..

[19] Li QY, Young B (2023). Experimental and numerical studies on cold-formed steel battened columns. Eng. Struct..

[20] AISI S-100 *North American specification for the design of cold-formed steel structural members* (AISI Standard, 2016).

[21] EN 1993–1–3: *Eurocode 3: Design of steel structures. Design of steel structures. Part1–3: General rules – Supplementary rules for cold-formed members and sheeting* (European Committee for Standardization, 2006).

[22] Roy K, Ting TC, Lau HH, Lim JB (2018). Nonlinear behaviour of back-to-back gapped built-up cold-formed steel channel sections under compression. J. Constr. Steel Res..

[23] Anbarasu M, Dar MA (2020). Axial capacity of CFS built-up columns comprising of lipped channels with spacers: Nonlinear response and design. Eng. Struct..

[24] *ABAQUS User's Manual, Version 6.14* (Hibbit, Karlsson, and Sorenson, Inc., 2004).

[25] Dabaon M, Ellobody E, Ramzy K (2015). Experimental investigation of built-up cold-formed steel section battened columns. Thin-Walled Struct..

[26] Schafer BW, Pekoz T (1998). Computational modelling of cold-formed steel: Characterizing geometric imperfections and residual stresses. J. Constr. Steel Res..

[27] Croccolo D, De Agostinis M, Vincenzi N (2011). Failure analysis of bolted joints: Effect of friction coefficients in torque—Preloading relationship. Eng. Fail. Anal..

